# A Longitudinal Study of Posttraumatic Stress Disorder Symptoms and Its Relationship with Coping Skill and Locus of Control in Adolescents after an Earthquake in China

**DOI:** 10.1371/journal.pone.0088263

**Published:** 2014-02-07

**Authors:** Weiqing Zhang, Hui Liu, Xiaolian Jiang, Dongmei Wu, Yali Tian

**Affiliations:** 1 West China School of Nursing/West China Hospital, Sichuan University, Chengdu, Sichuan Province, China; 2 Rui Jin Hospital, Shanghai Jiao Tong University School of Medicine, Shanghai, China; 3 West China Second Hospital, Sichuan University, Chengdu, Sichuan Province, China; Max Planck Institute of Psychiatry, Germany

## Abstract

**Background/Objectives:**

Post-traumatic stress disorder is a common psychological maladaptation among adolescents after undergoing an earthquake. Knowledge about the prevalence and maintenance of post-traumatic stress disorder symptoms and the changes of its predictors over time can help medical providers assist adolescent survivors with mitigating long-term impacts. This study examined the changes in posttraumatic stress disorder symptoms and its relationship with coping skill and locus of control among adolescent earthquake survivors in China.

**Methodology/Findings:**

The study used an observational longitudinal design. A total of 1420 adolescents were evaluated twice after the earthquake by using the Post-traumatic stress disorder Checklist-Civilian Version, The Internality, Powerful others and Chance scale and the Coping Styles Scale. The results indicated that the mean scores of posttraumatic stress disorder symptoms were decreased significantly and the positive rates of posttraumatic stress disorder symptoms also declined remarkably at 17 months compared to the 3 months post-earthquake. Internality locus of control and problem solving coping skill were effective resilient factors for the development and maintenance of posttraumatic stress disorder symptoms, while chance locus of control was a powerful risk factor of posttraumatic stress disorder symptoms as well as being female, being injured and property loss.

**Conclusions/Significance:**

Continuous screening is recommended to identify adolescent earthquake survivors with posttraumatic stress disorder symptoms. More attention should be paid to adolescent survivors who are prone to adopt passive coping strategies responding to trauma events and who own external causal attribution.

## Introduction

Catastrophic earthquakes are seemingly occurring more and more frequently around the world. Some earthquakes can lead to considerable losses in economic and social property, as well as create unaccountable personal or emotional loss. Posttraumatic stress disorder (PTSD) is a relatively common and complex psychiatric disorder experienced by earthquake survivors. PTSD is characterized by three symptom clusters that include re-experiencing, avoidance and numbing, and increased emotional arousal [1]. Several studies suggest that PTSD symptoms are common among earthquake survivors, and powerful earthquakes that cause thousands of casualties can have long-term physical and psychological consequences on survivors [2]–[4]. Nevertheless, few studies have examined the longitudinal course of PTSD among earthquake survivors [5], [6], especially adolescent survivors [7], [8].

Adolescents are more vulnerable to traumatic events and prone to develop psychiatric maladaptation. This is because adolescents are maturing in a way in which they must simultaneously deal with significant changes to their physical and psychosocial development [9], [10]. Growing evidence suggests that PTSD symptoms are common among adolescent earthquake survivors, and large variations in the prevalence of PTSD have been found in the wake of earthquakes: from 21.7% to 70.3% [11]–[13]. Some epidemiological studies have revealed several risk factors for developing psychological problems post-earthquake, including being female [14], [15], proximity to the epicenter [16], death or severe injury to family members [17], serious destruction of property/home [15], [18], degree of exposure to earthquake [6], [19], and less material and psychological support [15]. It is generally accepted that people cope with trauma in different ways [20]. Research has indicated that not everyone who experiences the same traumatic event develops PTSD or other psychiatric disorders [21]. Thus, coping styles and causal attributions might influence the development of posttraumatic psychological morbidity. For instance, among a group of individuals exposed to a traumatic event, coping styles of the individual and family were highly correlated with the occurrence of PTSD rather than the extent of injury [22]. A prior study in our lab also found that coping strategies and locus of control were significantly correlated with adolescents’ PTSD symptoms three months after an earthquake [15].

Although a few previous studies have addressed PTSD prevalence among adolescent earthquake survivors, to our knowledge, fewer studies have explored longitudinal changes in PTSD symptoms among adolescents, as well as changes in predictors for PTSD. Such information is important for school nurses and other health care professionals for early resiliency intervention to prevent or reduce adolescents’ psychiatric distress. On March 12, 2008, the Wenchuan earthquake, measuring 8.0 on the Richter scale, hit a densely populated province in southwest China. The earthquake resulted in several deaths, injuries, and significant property damage. At least 5,335 children and adolescents died in the earthquake, and several schools were destroyed.

Therefore, the aims of this study were to examine the longitudinal development of adolescents’ PTSD symptoms, changes in risk factors likely to predict adolescents’ PTSD symptoms, and the relationship between PTSD symptoms, coping strategies, and locus of control following the earthquake.

## Materials and Methods

### Ethics Statement

The present study was approved by the Human Subjects Ethics Sub-committee of Sichuan University. The approval of the Ethics Sub-committees includes consent procedure and written informed consent form. The written informed consents were obtained from all participating adolescents in the classroom by researchers, and verbal informed consents were obtained from the parents or the legal guardian of the involved adolescents through telephone.

### Samples

The current study adopted an observational longitudinal design and was carried out in five of the most severely damaged middle schools around the earthquake’s epicenter. All students conforming to the following sample criteria were recruited: 1) the students went through the earthquake personally; 2) both the student and his/her parents or guardians agreed to participate; and 3) the age of the students ranged from 12–20 years old.

### Data Collection

Data were collected using a self-report questionnaire that includes Post-traumatic stress disorder Checklist-Civilian Version, The Internality, Powerful others and Chance scale and the Coping Styles Scale. First time data collection was at 3 months after the earthquake. And the participants who have accepted participation in the follow-up investigation were reassessed 14 months later. The baseline evaluation comprised demographic data, earthquake exposure features, internal characteristics and PTSD symptoms, while the follow-up study consisted of demographic data and PTSD symptoms.

### Instruments

Basic information was obtained by a questionnaire consisting of demographic characteristics (e.g., age and gender), as well as features of earthquake exposure and internal characteristics of the subjects. Features of earthquake exposure included physical injury, self-exposure to burial and/or amputation, death of a family member(s), death of classmates or friends, witness to injury, witness to burial and/or death, and the degree of property loss. Internal characteristics of the subjects consisted of two scales measuring coping strategies and locus of control, which are described in detail below.

#### Coping strategy

A native Coping Styles Scale, which was designed and widely used in China specifically for middle school students, was used to assess coping strategies. The instrument is a 30-item self-administered scale with answers ranging from 1 (“I usually don’t do that at all”) to 5 (“I nearly always do that”). The scale includes six kinds of coping skills including problem-solving skill, resorting, withdrawing, abreacting, imagining, and tolerating. The explanation of each strategy has been described in our previous study [15]. The internal consistency (Cronbach’s α) values were 0.83, 0.71, 0.58, 0.51, 0.70, 0.48, and 0.77 for the problem-solving, resorting, withdrawing, abreacting, imagining, tolerating, and whole scale, respectively. The test-retest correlation coefficients ranged from 0.68–0.89 [23]. Higher scores indicated more frequent use of the corresponding coping strategy.

#### Locus of control

Locus of control was evaluated by the Internality, Powerful Others, and Chance scales developed by Levenson and Miller [24]. The scale is composed of 24 items and contains three subscales, including internality scale testing the degree to which a people has internal locus of control expectancies for the outcome of events, power others scale testing the degree to which a person believes that event outcomes are controlled by powerful others, and chance scale testing the degree to which a person believes that event outcomes are controlled by chance variables. The instrument is self-administered using a 7-point Likert scale, with answers ranging from −3 (“strongly disagree”) to +3 (“strongly agree”). The internal consistency values were 0.51, 0.62, and 0.70, for the source of internal control, powerful others control, and chance control, respectively [25]. Each subscale is calculated by summing all items belonging to that scale and adding 24 to counteract negative values and provide convenience for statistical analysis. For each subscale, the total score ranges from 0–48, with higher scores indicating a stronger belief in that particular source of control.

#### Posttraumatic stress symptoms

PTSD symptoms were evaluated by using The PTSD Checklist-Civilian Chinese Version (PCL-C) developed by the Behavioral Science Branch of the American Post-Trauma Stress Disorder Research Center, according to DSM-IV criteria, in 1994. The PCL-C is a 17-item self-report scale that evaluates PTSD re-experiencing, avoidance and numbing, and increased arousal category symptoms. The severity of symptom bothering during the past month is rated on a 5-point Likert scale, ranging from 1 (“none”) to 5 (“extremely”). Former study showed that diagnostic accordance rate between PCL-C and Clinician Administered PTSD Scale for DSM-IV (CAPS-DX), the gold standard for diagnosing PTSD, was 91.3% and misdiagnosis rate was 10%. And the internal consistent reliability of the whole scale was 0.82, and split-half reliability was 0.65. The test–retest reliability was good as well (r = 0.71, p<0.001; paired t = 0.44, p = 0.66) [26], [27]. A formal diagnosis of showing a positive corresponding symptom cluster is considered likely if participants endorse having been at least moderately bothered by (score ≥3) one or more re-experiencing symptoms, three or more avoidance symptoms, and two or more arousal symptoms. Summing all responses calculates a total score ranging from 17 (no symptoms) to 85, with higher scores indicating more severe PTSD symptoms.

### Data Analysis

SPSS 13.0 software was used for data analysis. Continuous data are presented as means, with standard deviations and quartiles where appropriate. For between-group comparisons, chi-square tests were used for categorical variables, and paired t-tests were used for continuous variables. Stratified stepwise multiple linear regressions were performed to examine the contribution of the independent variables to the total PCL-C score during both assessments. A p<0.05 (two-tailed) was considered significant.

## Results

### Demographic Characteristics, Earthquake Exposure Features, and Internal Characteristics

In total, 1,976 adolescents were evaluated three months after the earthquake, and among them, 1,420 were reevaluated 17 months after the earthquake. Those who did not participate in the second survey had graduated, transferred to another school, or refused to participate. In order to guarantee equivalent and unbiased sampling for further meaningful comparisons on the target variables in the present study, independent-sample t test was performed on age, scores of PTSD symptoms and locus of control, and Chi-square test was adopted for comparison on gender and earthquake exposure between both assessing participants and missing participants. As shown in Table 1, it results in no difference except parents died and witnessed injury. Thus the samples have good equivalent and representativeness and further group comparisons on PTSD symptoms and the relationship among PTSD symptoms, coping skills and locus of control were eligible.

**Table 1:** Description of demographic characteristics, earthquake exposure features and internal characteristics in adolescents after the earthquake.

	Both Assessing Participants n = 1420		Missing Participants n = 556	
Variables	Freq.	Pert.(%)	Freq.	Pert.(%)
***demographic characteristics***				
Age (M±SD)	15.77	1.149	15.65	.894
Gender				
Boy	604	42.5	234	41.3
Girl	816	57.5	332	58.7
***Earthquake Exposure***				
Buried	35	2.5	19	3.4
Injured	128	9.0	62	11
Amputated	10	0.7	3	0.5
Parents died	37	2.6	6	1.1*
Classmates died	544	38.3	220	38.9
Friends died	511	36.0	185	32.7
Witnessed burial	474	33.4	167	29.5
Witnessed death	508	35.8	220	38.9
Witnessed injury	878	61.8	428	75.6*
Property loss				
Mild	68	4.8	32	5.7
Moderate	111	7.8	53	9.4
Severe	386	27.2	134	23.7
Extremely severe	855	60.2	347	61.3
**Internal Characteristics**	M	SD	M	SD
***Coping styles***				
Problem Solving	3.27	.586	3.28	.595
Resorting	2.77	.624	2.69	.604
Withdrawing	2.89	.570	2.94	.543
Abreacting	2.53	.731	2.66	.723
Imagining	2.67	.880	2.63	.834
Tolerating	3.17	.680	3.10	.611
***Locus of control***				
Internality	28.57	6.911	28.73	6.919
Power others	15.78	9,176	15.05	9.049
Chance	18.71	7.693	18.17	7.523

*p<0.05 by chi-square test.

Of the 1,420 students, 42.5% were boys, and the mean age of the sample was 15.77 (SD = 1.15). With regard to earthquake exposure, 9.0% were injured, and 61.8% witnessed people injured in the earthquake. Some students also experienced people around them die in the earthquake. The percentage of students who lost parents, classmates, and friends were 2.6%, 38.3%, and 36.0%, respectively. Moreover, all students indicated that their family suffered varying degrees of property loss. More features of earthquake exposure and scores on the coping skills and locus of control measures are displayed in Table 1.

### Changes in Prevalence and Severity of PTSD Symptoms

Table 2 shows PTSD symptom scores, as well as the total PCL-C scores, among adolescents. Changes in severity of PTSD symptoms between both time assessments using paired t-tests are also reported. The mean total PCL-C score and scores on the three PTSD symptom clusters all decreased at re-evaluation. The mean total PCL-C score at 17 months (mean = 28.47, SD = 10.648) was significantly lower than at three months (mean = 33.24, SD = 10.657; t(1,419) = −24.397, p<0.001). In addition, the positive rates of PTSD symptoms showed the similar decline in severity of PTSD symptoms, 16.9% of adolescents were positive for all three symptom clusters at three months; this rate significantly decreased to 12.1% at 17 months, χ^2^ (1, N = 1,420) = 13.128, p<0.001. More changes of scores and positive rates of three PTSD symptom clusters are presented in Table 2.

**Table 2:** Difference analysis on scores and positive rates of PTSD symptoms in adolescents between both two times assessments after the earthquake (n = 1420).

		3-month		17-month			
Measures	Scores range	M	SD	M	SD	t value	p
**PCL-C**							
Re-experiencing	5–25	9.75	3.284	8.34	3.286	−106.337	.000
Avoidance & Numbing	7–35	12.45	4.803	10.91	4.643	−20.808	.000
Increased arousal	5–25	11.04	4.257	9.22	4.027	−19.717	.000
Total score of PCL-C	17–85	33.24	10.657	28.47	10.648	−24.397	.000
		Freq.	Pert.(%)	Freq.	Pert.(%)	χ^2^ value	p
Re-experiencing		851	59.9	555	39.1	123.415	.000
Avoidance and numbing		317	22.3	233	16.4	15.534	.000
Increased arousal		690	48.6	461	32.5	76.610	.000
All three symptom clusters		240	16.9	172	12.1	13.128	.000

### Predictors of PTSD Symptoms at Three Months and 17 Months after the Earthquake: Regression Analyses

Table 3 presents the results of stepwise regression analyses to determine factors that significantly predicted total PCL-C score at three months and 17 months post-earthquake. The independent variables included demographic characteristics, features of earthquake exposure, coping styles, and locus of control. In total, 12 independent variables were included in the regression analyses. Among them, chance locus of control was the most powerful predictor of PTSD symptoms at three months (β = 0.227, p<0.001) and the second most powerful predictor of PTSD symptoms at 17 months post-earthquake (β = 0.130, p<0.001). Female gender was the most powerful negative predictor of PTSD symptoms at three months post-earthquake, and problem-solving coping skill (β = −0.080, p<0.01) was the strongest predictor of PTSD symptom resilience at 17 months.

**Table 3:** The changes in predictors of total score of PCL-C by using stepwise multiple regression analysis in adolescents between both two times assessments after the earthquake (n = 1420).

		3-month		17-month	
Dependent variable	Independent variables^a^	B	Beta^b^	B	Beta^b^
Total Scores of PCL-C	Constant	14.345		23.985	
	Injured	1.863	.052*	4.762	.128***
	Classmates died	–	–	1.342	.061*
	Gender	−1.464	−.068**	−1.977	−.092***
	Property loss	1.027	.060*	.963	.249***
	Witnessed death	1.940	.088***	–	–
	Friends died	1.828	.085***	–	–
	Parents died	–	–	3.460	.093***
	Buried	–	–	3.910	.057*
	Witnessed injury	–	–	1.214	.055*
	Chance	.306	.227***	.180	.130***
	Power others	.181	.145***	.112	.096**
	Internality	−.099	−.060*	−.124	−.080**
	Problem solving	–	–	−1.760	−.097***
	Imagining	.685	.055*	1.063	.088***
	Abreacting	1.690	.107***	–	–
	Withdrawing	−1.352	−.057*	–	–
	Tolerating	.927	.056*	–	–
	F		33.177***		22.582***
	Adjusted R^2^		.214		.165

a variable assignment: gender (coded as girl = 0 or boy = 1), injured (coded as 0 = no or 1 = yes), buried (coded as 0 = no or 1 = yes), friends died (coded as 0 = no or 1 = yes), parents death (coded as 0 = no or 1 = yes), classmates death (coded as 0 = no or 1 = yes), witnessed injury (coded as 0 = no or 1 = yes) and property loss (coded as 1 = mild, 2 = moderate, 3 = severe or 4 = extremely severe).

b standardised regression coefficient.

*p<0.05; ** p<0.01; *** p<0.001.

## Discussion

Previous research has provided valuable information regarding PTSD symptoms and their risk factors in adolescence. However, few studies have employed longitudinal designs with large, representative samples focusing on the important role of internal features related to PTSD, such as coping skills and locus of control. The present study addressed these limitations and investigated prospective changes in PTSD symptoms and factors associated with changes over time.

Consistent with prior studies, we observed that overall adjustment of PTSD symptoms among adolescents presented a positive tendency. One longitudinal study investigating the course of PTSD found that the severity of PTSD symptoms among adolescents was lower at 32 months compared to three months after an earthquake [8]. Sahin et al. (2007) also reported that PTSD symptoms significantly decreased three months after an initial assessment post-earthquake. Chen and Wu (2006) observed changes in symptoms among students at one and two years post-earthquake, where the prevalence of all PTSD symptoms significantly decreased. The adolescents in the present study demonstrated decreased PTSD symptoms at 17 months as compared to three months post-earthquake. Furthermore, the positive rates of PTSD symptoms also declined significantly over time. The present findings might have resulted from the relatively good living conditions and effective social and health recovery programs implemented by the government and other health care providers. Previous studies have verified the benefit of timely social support, which could alleviate the psychological impact of an earthquake [28]. Certainly, there is also the possibility that symptoms could spontaneously improve as time passes [29]. However, the proportion of students who continued to experience PTSD symptoms 17 months after the earthquake was not less than expected. For instance, the percentage of students experiencing all three PTSD symptom clusters decreased by only 4.8% at 17 months. This confirmed our anticipated concerns that symptoms might persist long after a traumatic event and could become chronic. These findings highlight the necessity of long-term recovery efforts for improving the severity of PTSD symptoms experienced by adolescents, especially those who display positive PTSD symptoms.

An important aspect of the present study included an examination of coping skills and locus of control in the development of PTSD symptoms over time. We observed that “abreacting” and “tolerating” coping skills were significant risk factors of PTSD at baseline evaluation, and an “imagining” coping skill was a strong contributor to the severity of PTSD symptoms at both assessments. These three kinds of coping skills were considered as passive coping strategies. Previous studies support the notion that passive coping strategies are an adverse means of dealing with psychological maladaptation, leading to poor mental experience and posttraumatic stress symptoms. Conversely, problem-solving coping skills, such as positive thinking or actively dealing with problems, are associated with good adaptation to trauma. For instance, some positive coping strategies are correlated with decreased morbidity of psychiatric disorders after a traumatic event (i.e., in the aftermath of a terrorist attack; [30], [31]. The present study found that problem-solving skill was a negative predictor of PTSD symptoms at 17 months post-earthquake and helped mitigate the severity of PTSD symptoms. However, two interesting changes need to be clarified. Firstly, “withdrawing” skill as a classic passive coping strategy was a protective factor against PTSD symptoms at three months but was not predictive of PTSD symptoms 17 months post-earthquake. Previous studies found that passive coping skills might be useful in the short term after a traumatic event because they help survivors defend against formidable mental assault; however, if this defensive style obstructs overcoming of the trauma, then the likelihood of maladaptation increases. [32]. The second change was that the problem-solving coping skill was a negative predictor of PTSD at 17 months but not at three months post-earthquake. This change highlights that coping should be seen as a process, and individuals choose a coping style depending on the situation with which they are confronted. As time went on, and the stressor changed, adolescent survivors chose the strategy they considered most effective to deal with their problems [33]. The problem-solving coping strategy could be regarded as an effective means for adapting to stressors and fighting against hazards resulting from traumatic events in order to prevent the development of chronic PTSD.

Locus of control refers to the extent to which individuals believe that they can control events that affect them. A person’s locus is conceptualized as either internal (the person believes they can control their life) or external (meaning they believe that their decisions and life are controlled by environmental factors which they cannot influence). Social psychologists have discovered the correlation between external locus of control and maladaptive behaviors. Contrarily, prior studies found that subjects with an internal locus of control are more zealous in seeking after solutions to their problems. However, the relationship between locus of control and PTSD symptoms in children or adolescents was rarely reported. And March et. al. (1997) found similar results that an external locus of control associated with female children were prone to develop PTSD aftermath of the Hamlet fire [34]. The results of the present study showed that locus of control explained a significant amount of variance in PTSD symptoms, above and beyond earthquake exposure. This suggests an important role of locus of control in the development of PTSD among adolescents experiencing earthquake-related trauma. Consistent results at both assessments were observed whereby chance locus of control and powerful others locus of control were strong risk factors for PTSD symptoms. Conversely, an internal locus of control was a strong protective factor against PTSD symptoms. Individuals with an internal locus of control perceive events being contingent on their own behavior. Compared with the chance and powerful others locus of control, an internal locus of control promotes positive health outcomes because this control style is associated with preventive behaviors, efforts to improve functioning, and useful resilience to psychological dysfunction [35], [36]. Conversely, people with an external locus of control believe that events occur beyond their control, such as fate, chance, or powerful others. These individuals do not fight to protect themselves when suffering from psychological harm, and they believe that they cannot change the situation because someone/something else is in control or they are destined to encounter the traumatic event. Consequently, it is easy to comprehend that students with a more external locus of control were more likely to develop PTSD symptoms. This suggests that an optimistic causal attribution style for positive outcomes is associated with better psychological adaptation.

Additionally, the present study identified other risk factors for the development and maintenance of PTSD symptoms, including being female, being injured, property loss, and so on. Similar findings have been reported elsewhere [15]–[18].

## Conclusions

Results of the current study provide useful information that enriches our knowledge of the developmental trajectory of posttraumatic stress disorder symptoms and changes in posttraumatic stress disorder predictors over time. Furthermore, we observed that coping styles and locus of control contributed to the development and maintenance of posttraumatic stress disorder symptoms. In the short term post-earthquake, passive coping skills may protect adolescent survivors from psychological harm; however, active coping skills are propitious to adjustment in the long term. Additionally, an internal locus of control contributes to positive posttraumatic adjustment. In light of these findings, a couple of points need to be highlighted: First, adolescent survivors are vulnerable to posttraumatic stress disorder symptoms following an earthquake, which could develop into chronic posttraumatic stress disorder. Second, adolescent survivors prone to adopting passive coping strategies and have an external locus of control should be targeted for disaster intervention and health promotion support.

### Limitations and Suggestions for Future Studies

Some limitations of the present study should be noted. First, the instruments adopted were all self-reported; thus, information collected may have been over- or under-estimated. Second, our findings are specific to Chinese adolescents and might not be generalizable to other ethnic groups. Third, the present study only examined certain risk factors for posttraumatic stress disorder symptoms but other factors, which likely affect posttraumatic stress disorder symptoms (i.e., material or psychological support and school performance), warrant future investigation. Finally, a combination of quantitative and qualitative research methods is needed to provide much richer information on posttraumatic stress disorder symptoms among adolescent trauma survivors.
